# Dynamic propagation of an Airy beam in metasurface-enabled gradiently-aligned liquid crystals

**DOI:** 10.1515/nanoph-2023-0516

**Published:** 2023-10-30

**Authors:** Meini Gao, Jiawei Wang, Wenfeng Cai, Ming Cheng, Xichen Hao, Yuhan Wang, Ying Liu, Delai Kong, Jianxun Liu, Haitao Dai, Yan Jun Liu

**Affiliations:** Tianjin Key Laboratory of Low Dimensional Materials Physics and Preparing Technology, School of Science, Tianjin University, Tianjin 300072, China; Department of Electrical and Electronic Engineering, Southern University of Science and Technology, Shenzhen 518055, China; Shenzhen Engineering Research Center for High Resolution Light Field Display and Technology, Southern University of Science and Technology, Shenzhen 518055, China

**Keywords:** vectorial beam, optical linear potential, gradient refraction index

## Abstract

Due to the unique self-acceleration, self-healing, and non-diffraction properties, Airy beams have been explored extensively and found applications in various fields. It has been proven as an essential aspect to tune the trajectory of Airy beams for extensive applications. In this paper, we propose a method based on liquid crystal (LC) alignment with metasurfaces, which enables dynamic tuning of the trajectory of Airy beams. Benefiting from both the tunable property of LCs and the compact alignment of metasurfaces, we achieve a sizeable linear potential in a short distance, which leads to the effective tuning of the trajectory of Airy beams dynamically. The introduction of metasurfaces into the alignment of LCs provides a promising method to manipulate the planar optical field.

## 1 Introduction

Up to now, Airy beams have drawn intensive attention due to their unique properties of non-diffracting, self-healing, and self-accelerating along curved trajectories [1–23]. In recent decades, Airy beams have been widely used in plasma channels [5], particle manipulation [6, 7], atmospheric communication [8], laser-induced discharge [9], spatiotemporal photon bombs [10, 11], all-optical routing [12], and three-dimensional imaging [13–15]. Various methods have been developed to generate Airy beams, such as cubic phase masks [16, 17], designed optical metasurfaces [18, 19], asymmetric nonlinear photonic crystals [20], optical aberrations [21], and spatial light modulators (SLM) [22]. Of the most necessary is to tune the trajectory of Airy beams for broad applications. For example, the tunable trajectory offers an additional degree of freedom for atmospheric communication [8], all-optical routing [12], and 3D imaging [13–15]. Changing the generation parameters of the phase plate is a straightforward way to tune the trajectory [23, 24]. However, the change of the generation parameters will subsequently affect the other properties of Airy beams, such as the full-width at half-maximum (FWHM) of the main lobe. Another effective method is to manipulate the trajectory of Airy beams during their propagation, which is highly dependent on the medium properties. The propagation characteristics of Airy beams have been widely studied in various media, such as free space [2, 25, 26], nonlinear media [27, 28], inhomogeneous media [29, 30], chiral media [31], uniaxial crystals [32, 33], etc. A linear potential, which can be realized with the gradient refraction index (GRI) in the materials, has been proposed to effectively control the trajectory of Airy beams [34, 35]. Photorefractive crystals are a good candidate to achieve the GRI. Ye et al. [27] have demonstrated tunable Airy beams in strontium barium niobate (SBN) crystals, in which the GRI is formed by light irradiation with specially designed intensity distribution. However, only a small GRI (0.00045/mm) can be created in the photorefractive crystal at a very high driving voltage (4.2 kV/cm), which greatly limits practical applications. Therefore, it is highly desirable to find a new approach to dynamically control the trajectory of Airy beams.

For the GRI generation, liquid crystals (LCs) could be an ideal candidate, in which a significant refractive index modulation (0.2−0.3) can be achieved by controlling their alignment. Due to their large birefringence and versatile driving methods, LCs have been used in a wide range of applications, including Pancharatnam–Berry (PB) cubic phase masks [16, 17], smart windows [36], vector optical elements [37], metalens [38], metamaterial optical devices [39–43], biosensors [40], and lasers [44–46]. For LC-based devices, the alignment of LCs is a key factor that affects the performance of devices. Currently, mechanical rubbing and photoalignment are the prevailing methods for LC alignment. In contrast with mechanical rubbing, photoalignment is a noncontact alignment technique that eliminates the drawbacks of mechanical rubbing, such as surface damage, dust and electrostatic charge generation. It can be conveniently used to fabricate various optical elements with complex patterns, such as depolarizer [47], optical vortices generation [48], vector beams generation [49], polarized Airy masks [50], PB q-plates, and PB grating [51]. Moreover, using the photoalignment technique, one can create arbitrary patterns with a high resolution. For example, complex alignment patterns with a resolution of ∼1 μm have been demonstrated using a digital micro-mirror device (DMD) [47–50] or a laser direct-writing system [51, 52]. Even higher resolution can be achieved using laser interference [53, 54] and plasmonic photopatterning [55, 56]. However, the photoalignment technique is still limited by the stability issue and the highly demanded resolution (i.e., 1 μm). Recently, nanopatterns have been exploited for LC alignment in nanoscale resolution, such as atomic force-scribed nano grooves (120−300 nm) [57], lithographic metasurfaces (200−300 nm) [58], imprinted nanostructures (25 nm) [59], etc. More importantly, arbitrary nanopatterns with high-precision alignment capability can be fabricated, which is particularly favorable for the GRI generation.

This work proposes a method consisting of Au metasurfaces and nematic LCs, which can be used to achieve a linear potential to manipulate the trajectory of 1D Airy beams. Besides, the linear potential can be modulated with an external electric field resulting in the modulation of different trajectories. The tunable parabolic trajectory of Airy beams would stimulate novel applications, such as optical logical circuits, etc. Meanwhile, this work also provides a promising method for planar photonics [60, 61], which will trigger more exciting studies and applications.

## 2 Theory

As reported, the one-dimensional paraxial wave equation can be used to describe the propagation of Airy beams in LCs [3, 28]:

(1) $$\frac{\partial^{2}}{\partial x^{2}}\psi \left(x,z\right)+2ik\frac{\partial }{\partial z}\psi \left(x,z\right)+\frac{2k^{2}\Delta n}{n_{0}}\psi \left(x,z\right)=0,$$

where $\psi \left(x,z\right)$ is the wave function, *k* is the wave vector and is given by *k* = 2*πn*/*λ*_0_, and Δ*n* is refractive index change caused by LCs. To achieve a finite energy Airy function, Siviloglou et al. proposed an exponentially decaying form as follows [3]:

(2) ψ(s,ξ=0)=Ai(s)exp(as).

In Eq. (2), *s* = *x*/*x*_0_ represents a dimensionless transverse coordinate, $\xi =z/kx_{0}^{2}$ is normalized propagation distance, and *x*_0_ is an arbitrary scale factor. In addition, *a* is a positive quantity to ensure containment of the infinite Airy tail and enable the experimental realization of Airy beams. Equation (1) admits the Airy nondispersive solution with the initial conditions of Eq. (2). Nonlinear effect will be not prominent until the power is high enough [62]. When the nonlinear effect dominates the propagation of Airy beams in LCs, the parabolic trajectory would be disturbed drastically [63, 64], such as the formation of breathing soliton [65–67], etc. The birefringence of LCs would be enough to describe the properties with a power lower than the nonlinear effect threshold of light to LCs. At the same time, birefringence could be used to form an optical linear potential. A linear gradient of refractive index along the *x*-axis can be expressed as [28]:

(3) $$\Delta n=\delta_{n}x,$$

where *δ*_*n*_ represents the GRI along the *x*-axis. Considering Eqs. (1)–(3), the acceleration *g*_*x*_ of Airy beams in LCs can be deduced [28]:

(4) $$g_{x}=\frac{1}{4k^{2}x_{0}^{3}}+\frac{\delta_{n}/n}{2},$$

where *n* is the refractive index of the medium. According to Eq. (4), the transverse acceleration of Airy beams in LCs is proportional to the GRI. As known, the effective refractive index of LCs *n*_eff_ is related to the alignment angle of LCs and can be described as:

(5) $$n_{\text{eff}}\left(\theta \right)=\frac{n_{e}n_{o}}{\sqrt{\left(n_{e}^{2}-n_{o}^{2}\right)\sin^{2}\theta +n_{o}^{2}}},$$

where *n*_*o*_ and *n*_*e*_ are the ordinary and extraordinary refractive indices of nematic LCs, and *θ* is an angle between the director of LCs and the *z*-axis. The incident light propagates along the *z*-axis as well. Therefore, the GRI can be prepared with the gradient alignment of LCs, and subsequently used to tune the trajectory of Airy beams.

## 3 Materials and methods

### 3.1 Fabrication of LC samples

#### 3.1.1 Nanofabrication of metasurfaces

The gold metasurface was fabricated on an ITO-coated glass substrate utilizing electron-beam lithography (EBL), followed by a metal evaporation and lift-off process. The ITO-coated glass substrate was cleaned with acetone and isopropyl alcohol (IPA) in an ultrasonic bath. To pattern the gold metasurface via EBL, a positive electron beam resist (AR-P 6200.09) was spin-coated on the ITO-coated glass substrate to form a resist layer with a thickness of 150 nm. It was prebaked at 150 °C for 2 min. To prevent the charge accumulation effect during subsequent electron beam exposure, a thin layer of conductive solution (AR-PC 5090.02) was spin-coated. EBL was carried out with a system (nB5, Nano beam). The current and voltage used were 4 nA and 80 kV, respectively. Each pattern exposed subfield was 20 × 20 μm^2^. The pattern development was done in AR 600–546 for 60 s. A 2 nm chrome adhesion layer and a 50-nm-thick gold film were subsequently deposited on the resist pattern, in an electron beam evaporation system (TF-500, HHV). The evaporation rate during the deposition process was controlled to be ∼0.4 Å s^−1^ with the vacuum level of 5 × 10^−6^ Torr inside the evaporator chamber. Finally, the gold metasurfaces were achieved after a lift-off process in the resist remover (AR 600–71).

#### 3.1.2 Fabrication of the LC cell

Once the fabricated metasurfaces were ready, two pieces of glass substrates were mirror-symmetrically arranged in the upper and lower to assemble a LC cell. Alignment markers are used to align the two pieces of substrates under the microscope. The thickness of the LC cell was controlled to be 100 µm to facilitate the coupling of the free-space Airy beam. The substrates were assembled to form an LC cell with an optical adhesive NOA65 and then UV-cured for 5 min. Upon the LC cell ready, LCs (E7, *n*_*o*_ = 1.52 and *n*_*e*_ = 1.7032) were infiltrated at 70 °C by the capillarity force and then cooled down naturally.

### 3.2 The experimental setup

**Figure 1:**
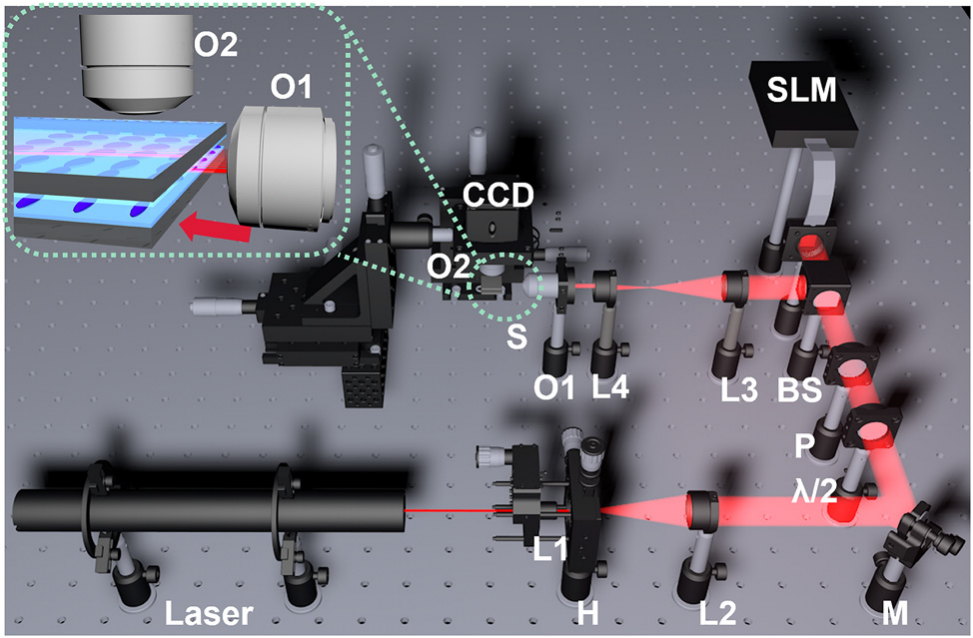
Schematic experimental setup for generation and recording the propagation trajectory of Airy beams. M: mirror, H: pinhole, L: lens, *λ*/2: *λ*/2 plate, P: polarizer, BS: beam splitter, SLM: spatial light modulator, O: objective lens, CCD: charge-coupled device, S: sample.

The cubic phase method is used to generate free-space Airy beams, as reported [2, 3, 66]. The experimental setup is schematically shown in Figure 1. The emitting light from the He–Ne laser at *λ* = 632.8 nm is impinging on the surface of the spatial light modulator (SLM, HOLOEYE Photonics AG, Germany) after spatial filtering, expanding (L1, H) and collimating (L2). The focal lengths of L1 and L2 are 4.51 mm and 200 mm. Before reaching SLM, a half-wave plate (*λ*/2) and a linear polarizer (P) are used to ensure the polarization direction of light parallel to the axis of LC alignment in SLM. The SLM-modulated light is reflected by the beam splitter (BS) towards the sample and CCD camera. A 4*f* system (L3: *f* = 250 mm, L4: *f* = 25 mm) and an objective lens (O1, 40×) are exploited to reduce the light beam size to nearly 100 μm and perform the Fourier transform. The generated Airy beam is then coupled into the LC layer along the direction indicated by the red arrow, as shown in the inset in Figure 1. In the direction perpendicular to the substrate, a CCD camera in combination with an objective (O2, 10×) is used to record the trajectory of the Airy beam’s propagation in the LC layer.

## 4 Results and discussion

### 4.1 Numerical simulation

**Figure 2:**
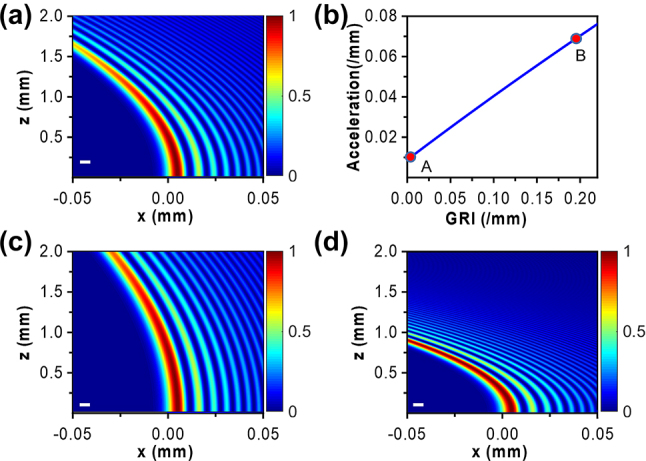
The propagation trajectories of Airy beams in air (a) and in LCs (b–d) with the GRI. (b) The theoretically calculated acceleration of the Airy beam as a function of the GRI. The corresponding accelerations are 0.0203/mm (in air), 0.0088/mm [in LCs with GRI of 0/mm, corresponding to dot A in (b)], and 0.0714/mm [in LCs with GRI of 0.2037/mm, corresponding to dot B in (b)]. The scale factor *x*_0_ = 5 µm of Airy beams was used for calculation.

As described theoretically, the one-dimensional paraxial wave equation can be used to describe the propagation of Airy beams in LCs. The gradient alignment of LCs can create the GRI, which can be further used to tune the trajectory of the finite energy Airy beam. The propagation trajectories of an Airy beam with and without the linear potential are depicted in Figure 2. The trajectory of the Airy beam in air is shown in Figure 2a. Correspondingly, the calculated acceleration of the Airy beam is 0.0203/mm. Figure 2b shows the acceleration of the Airy beam as a function of GRI, demonstrating that the acceleration increases with an increasing GRI in theory. The marking dots A and B in Figure 2b represent two cases in the LC cell with GRI of 0/mm and 0.2037/mm, respectively. Figure 2c and d illustrate the corresponding cases of the marking dots A and B. In Figure 2c, LC molecules are uniformly aligned with the director parallel to the *z*-axis (i.e., the propagation direction of the incident light). The calculated acceleration of the Airy beam is 0.0088/mm. While in Figure 2d, the director of LCs gradually changes from 0° to 80° along the *x*-axis, possessing GRI of 0.2037/mm. Correspondingly, the calculated acceleration of the Airy beam is 0.0714/mm. It is also noted that the FWHM (8.3 µm) of the main lobe keeps unaltered. The simulation parameter *x*_0_ = 5 µm for Airy beams is reasonably used for the observation in the following experiment.

### 4.2 Design and realization of the optical linear potential

**Figure 3:**
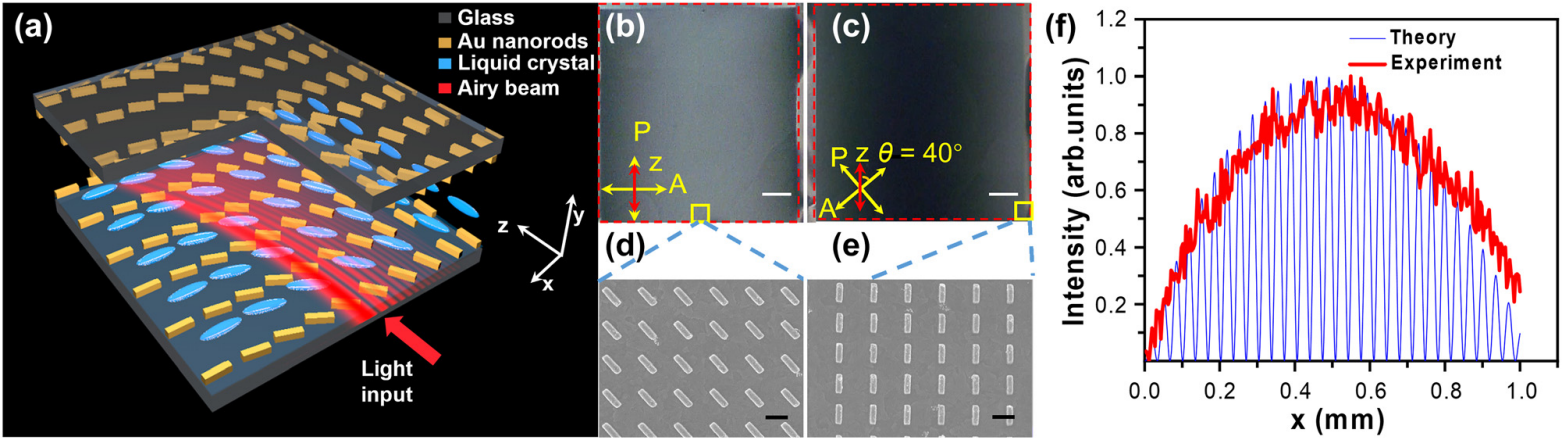
Design and characterization of the optical linear potential. (a) Schematic diagram of the LC cell with gradient LC alignment enabled by the metasurface. The red arrow indicates the propagation direction of the Airy beam. (b, c) POM images of the LC cell with the GRI under different polarization configurations, (b) *θ* = 0○ and (c) *θ* = 40○, where *θ* is the angle between the optical axis of polarizer P and the *z*-axis. (d, e) SEM images in the middle (d) and at the edge (e) of the fabricated metasurface labeled as yellow squares in (b & c). (f) Theoretically calculated (blue curve) and experimentally measured (red curve) transmission intensity distributions of the LC cell under the POM. P and A in (b & c) represent the optical axes of the polarizer and analyzer in the POM, respectively. Scale bar: 0.2 mm in (b & c) and 600 nm in (d & e).

As aforementioned, the GRI is an optical linear potential, which can be used to tune the trajectory of Airy beams. In this work, the GRI is generated by metasurface-aligned LCs. As reported [58], the LC molecules can be aligned by the gold-nanorod-based metasurface with their director along the long axis of the gold nanorods. Therefore, the effective index distribution of LCs can be achieved by programming the nanorod orientation of the metasurface. The designed gold-nanorod-based metasurfaces is on an indium-tin-oxide (ITO)-coated glass substrate, as shown in Figure 3a. The metasurfaces on both the upper and lower substrates are mirror-symmetrically arranged, ensuring to produce consistent alignment of the LC molecules from the top to the bottom across the LC cell, but gradually changed alignment along the *x*-axis to create the GRI. The metasurface structure has a period of *P* = 600 nm. Each gold nanorod has the length of *L* = 400 nm, the width of *W* = 100 nm, and the thickness of *T* = 50 nm. Moreover, we set 10 μm as a pixel, in which each gold nanorod has the same orientation. As shown in Figure 3a, the orientation angle of gold nanorods varies from 0° to 80° along the *x*-axis and keeps uniform along the *z*-axis, which ensures the index gradient 0.2037/mm of LCs (E7) along the *x*-axis. Upon the fabrication of metasurfaces, two pieces of glass substrates were carefully aligned with the help of aligning markers to assemble a LC cell and sealed with the UV-cured optical adhesive. Experimentally, the Airy beam will be coupled into the LC cell from the *x*–*y* plane and then propagate along the *z*-axis.

To confirm the LCs’ alignment quality by the metasurface, the aligned LC cell was investigated under the polarizing optical microscope (POM), as shown in Figure 3b and c. It can be seen that the observed colors are quite uniform along the *z*-axis, indicating that the LCs have the same alignment; while the observed colors become gradually changed from bright in the middle to dark at the edge (see Figure 3b) or from dark in the middle to bright at the edge (see Figure 3c) along the *x*-axis, indicating that the LCs’ alignment angle has gradual change. Figure 3d and e shows the magnified SEM images of the fabricated gold metasurface at different positions labeled in Figure 3b and c. Furthermore, the optical intensity distribution along the *x*-axis under the POM was detected and compared with the theoretical intensity distribution calculated by the Eq. (6) [68], as shown in Figure 3f. *α* and *β* are the angles between the optical axis of the polarizer/analyzer and the fast axis of LCs, respectively. *δ* is the maximum phase difference between the ordinary and extraordinary light upon passing through the LC cell.

(6) $$I=A^{2}\left[\cos^{2}\left(\alpha -\beta \right)-\sin^{2}\frac{\delta }{2}\sin2\alpha \sin2\beta \right]$$

It is obvious that the experimental intensity distribution has a good agreement with the theoretical intensity distribution. As a result, we can conclude that LCs can be well aligned by the designed metasurface and a linear potential is therefore achieved.

### 4.3 Electric field characteristics

**Figure 4:**
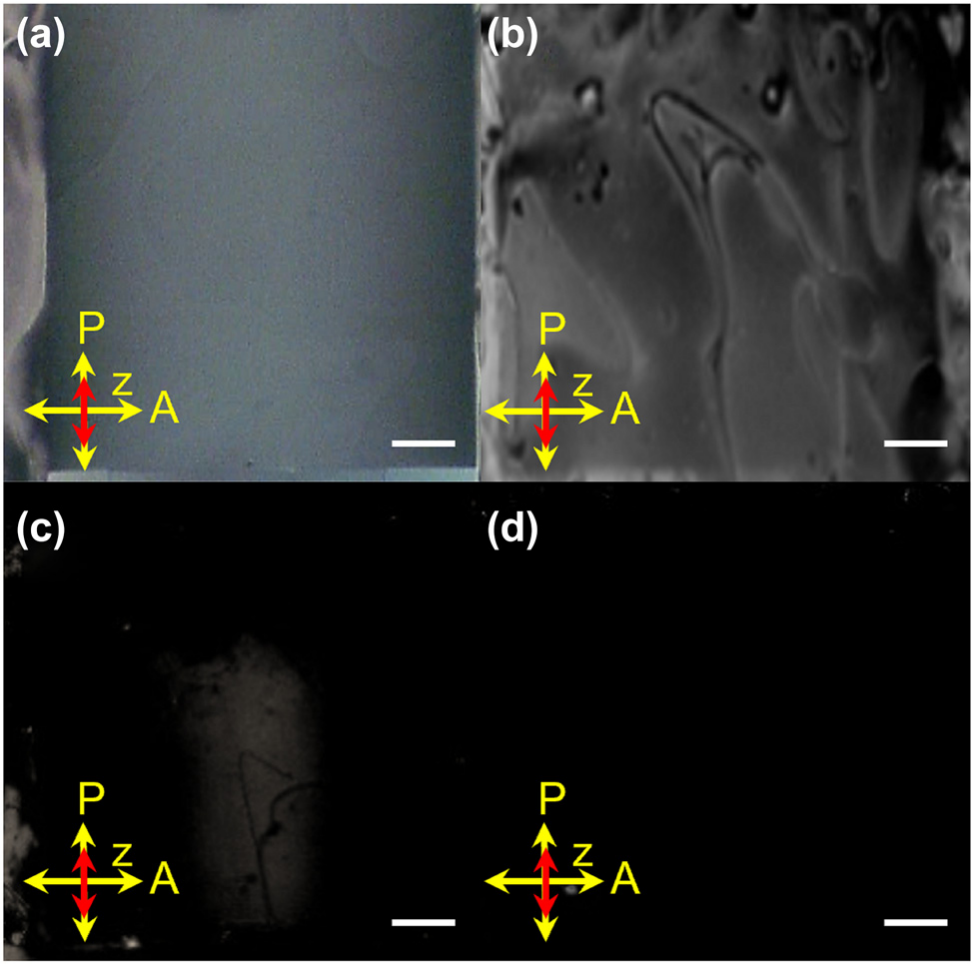
POM images of the LC cell with the applied voltages of (a) 0, (b) 100, (c) 200, and (d) 250 V_pp_, respectively. Scale bar: 0.2 mm.

As known, when the LC cell is applied a voltage, the created electric field will re-align the LC molecules, hence changing the GRI inside the LC cell. Therefore, the aligned LC cell was investigated under different applied voltages of 0, 100, 200, and 250 V_pp_, respectively, as shown in Figure 4. At *V* = 0 V_pp_, there is an apparent gradient intensity inside the LC cell, as shown in Figure 4a. At *V* = 100 V_pp_, the gradual alignment of LCs is completely destroyed, as shown in Figure 4b, indicating that the created GRI disappears at this state. As the voltage increases to 200 V_pp_, most of LC molecules are aligned along the electric field direction, as shown in Figure 4c, in which the GRI distribution inside the cell can’t be described simply. At *V* = 250 V_pp_, the POM image shows a completely dark view in Figure 4d, confirming that all the LC molecules can be re-aligned along the electric field direction. At this state, the coupled light will experience a uniform LCs with the refractive index of *n*_*o*_ = 1.52 and the GRI of 0/mm.

### 4.4 Dynamic propagation of Airy beams

**Figure 5:**
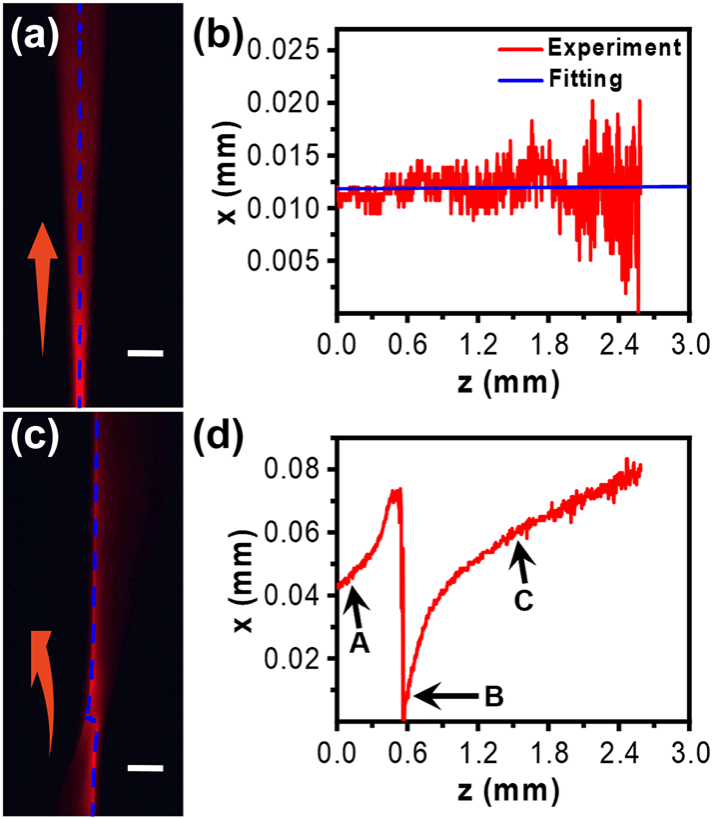
The CCD-recorded trajectories of the Gaussian beam (a) and Airy beam (c) in air. The blue dotted lines indicate the center and the main lobe of the light beams analyzed in (b) and (d), respectively. Red arrows indicate the beam propagation direction. Scale bar in (a & c): 0.3 mm.

With knowing the LC cell properties, we will then investigate the propagation and tuning properties of Airy beams inside the LC cell. The experimental setup in Figure 1 should be well calibrated. Firstly, a Gaussian beam was used to calibrate the experimental setup in our experiment. The trajectory of the Gaussian beam is provided in Figure 5a. The blue dotted line indicates the center of the beam. The fitting curve is shown in Figure 5b. The primary term coefficient is 7.5 × 10^−5^, which means the lateral displacement shall not exceed 1.5 nm after 2 mm propagating distance along the *z*-axis. Next, it is necessary to ensure that the experimentally generated Airy beam is consistent with the theoretical prediction. The trajectory of the Airy beam in air is fitted, as shown in Figure 5c. Figure 5d shows the propagation trajectory of the Airy beam in air. The blue dotted line indicates the center of the main lobe of the Airy beam. The main lobe and side lobe will alternate during propagation after the beam passes through the lens due to the Fourier transform. The trajectories A → B and B → C are not the same parabolas. It can be seen in Figure 5d, the trajectory from A → B point is the side lobe before the alternation of the main and the side lobe. Therefore, the main lobe was fitted for the trajectory between B and C point with the acceleration of 0.019/mm, which is very close to the theoretical value of 0.0203/mm given the experimental errors. After calibration, the theoretically designed Airy beams are generated for further investigation.

**Figure 6:**
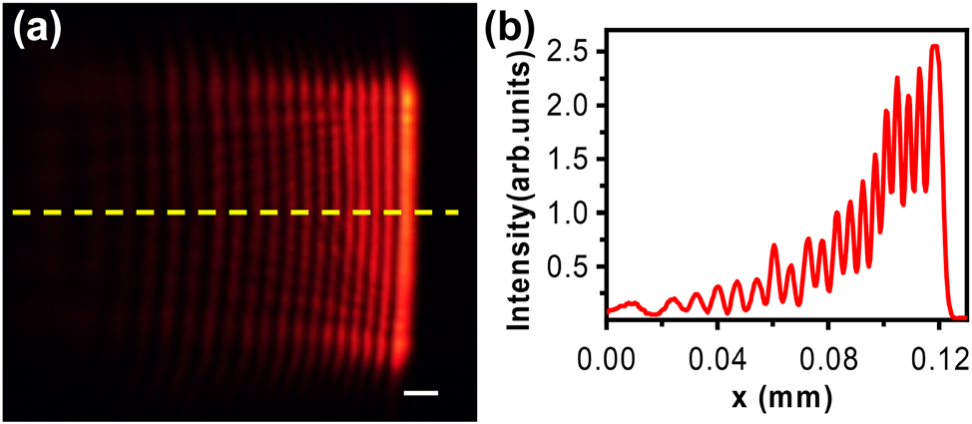
The field distribution in the *x*–*y* plane of Airy beams (a) and the intensity profile (b) along the line labeled in (a). Scale bar: 0.01 mm.

The trajectory of Airy beams inside the LC cell can be recorded and characterized. Before characterizing the properties of the Airy beam in liquid crystals, one of the most important things is making sure that the Airy beam is achieved as designed. Therefore, the cross-section of the Airy beam in the *x*–*y* plane in air and the intensity profile along the *x*-axis are shown in Figure 6. Figure 6a shows the cross-section of the Airy beam in the *x*–*y* plane in air, which presents the typical Airy oscillation. Figure 6b shows the intensity profile along the *x*-axis with the FWHM of 6.3 µm, which corresponds to the yellow dashed line in Figure 6a. As a result, the free-space 1D Airy beam is successfully generated in our experiment.

**Figure 7:**
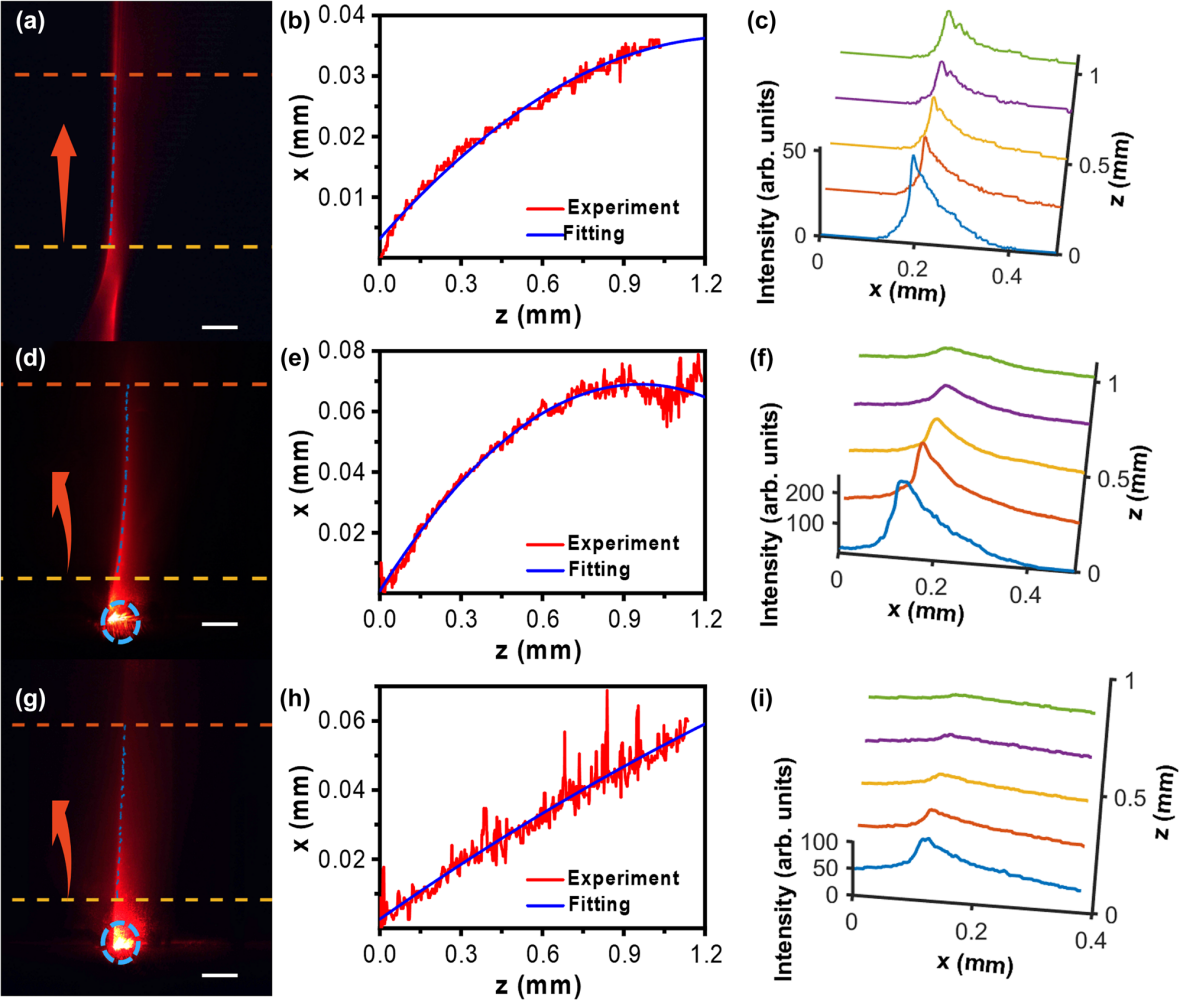
The propagation properties of Airy beams in air (a–c), and in LCs with GRI (d–f) and without GRI (g–i) including the images in *x*–*z* plane (a, d, g), the trajectory curves with both experimental measurement and numerical fitting (b, e, h), and cross-sectional intensity profiles at different propagation distances (c, f, i). The GRI inside the LC cell was 0.2037/mm. The GRI inside the LC cell is erased by applying a high enough voltage, resulting in the uniform ordinary index of LC (E7, *n*_0_ = 1.52) for the incident beam. Red arrows indicate the beam propagation direction. Blue circles label the starting point of beam coupling. Scale bar in (a, d, and g): 0.3 mm.

In our work, the distinct advantage is that the specially aligned LCs by the metasurface are used to tune the trajectory of Airy beams. Figure 7 depicts the effect of LCs on Airy beams. First, Airy beams propagating in air are presented in Figure 7a–c. Numerical fitting was carried out through the range between two dashed lines in Figure 7a. The experimentally achieved acceleration is 0.019/mm, which is in good agreement with the theoretical value of 0.0203/mm considering the experimental errors. The numerical fitting of main lobe and experimental results are in excellent agreement, as shown in Figure 7b. It was worth mentioning that in Figure 7a and b, only the beam trajectory between two dashed lines was considered due to the effective propagating distance of the Airy beam. As known, Airy beams are a type of diffraction-free beam for a limited propagation distance due to the apodization. The maximum propagating distance of being diffraction-free is determined by the generating parameters of the Airy beam [5]. The experimental parameters are set as: *λ* = 632.8 nm, *φ* = 20*π*, *x*_0_ = 0.005 mm. The effectively propagating distance can be deduced as below:

$$z_{0}=7.17\varphi_{\text{total}}^{1/3}x_{0}^{2}/\lambda .$$

The calculated effective distance of the Airy beam is 1.1261 mm. In Figure 7a, the distance between two dashed lines is about 1 mm, in which the Airy beam propagates without apparent diffraction, as shown in Figure 7c.

The side lobes are not clearly observed in Figure 7a–c, which might be caused by the weak scattering and intensity of side lobes. The more important reason is that the scale bar in Figures 5 and 7 is thirty times larger than the one in Figure 6, indicating that the magnification is thirty times lower. However, we have to sacrifice the resolution to observe and analyze the overall trajectory. As a result, the Airy beams’ side lobes appear less distinct under the observation with a low magnification. In our experimental setup, the trajectory of the Airy beams is retrieved by collecting the scattering light from the top of the LC cell by the CCD camera. Due to the weak intensity of side lobes and the weak scattering, the recorded image by the CCD is not very clear. Therefore, only the main lobes are considered in our experiments.

Figure 7d–i shows the trajectories of the main lobe of the Airy beam in the LC layer with and without the applied voltage. Figure 7d–g presents the recorded images of the Airy beam in the LC cell without and with the applied voltage, respectively. In Figure 7d, the main lobe obviously curves towards the left due to the linear potential. As aforementioned, the designed LC alignment forms a linear potential (0.2037/m) in the LC cell. The fitting acceleration of the Airy beam is about 0.074/mm, which is in good agreement with the theoretical value of 0.0714/mm, as shown in Figure 7e.

As theoretically predicted, the trajectory of Airy beams will become flattened by decreasing the linear potential. In our experiment, the linear potential can be decreased by applying a voltage to the LC cell. Figure 7g shows the recorded trajectory of the Airy beam when applying the high voltage of 250 V_pp_ with the frequence of 1 kHz. The LC molecules will be completely re-aligned along the electric field direction, leading to a zero GRI. As a result, the LC cell can be considered as a uniform media with the ordinary refractive index (1.52 for LC E7). Figure 7h describes the fitting curve of the Airy beam under this condition. The measured acceleration from the fitting curve is 0.0065/mm, which is very close to the theoretical prediction (0.0088/mm). The discrepancy between the experiment and theory might be caused by the light scattering in the LC cell. The results of Figure 7d–g confirm the effective modulation of the Airy beams in the LC cell, providing a promising method for optical routing and computing. Noting that Figure 7a–g presents the propagation trajectories of Airy beams in uniform media with the refractive index of 1 and 1.52, respectively. Their corresponding fitting accelerations are 0.019/mm in air and 0.0065/mm in the LC layer. Therefore, we can also conclude that a larger index causes a decrease of the acceleration for Airy beams, which agrees well with the theoretical prediction.

## 5 Conclusions

In this work, we have demonstrated that the propagation trajectory of Airy beams can be electrically manipulated in a metasurfaces-aligned LC layer. A linear potential (i.e., GRI) has been successfully achieved in the LC cell with specially designed metasurfaces. The propagation trajectories of Airy beams in LC cells with and without the GRI have been explored experimentally. The results show that the GRI of LCs can tune the main lobe of Airy beams effectively. The acceleration can be modulated from 0.0088/mm to 0.0714/mm, and more, which dramatically exceeds the photorefractive crystal. Furthermore, the electrically tunable property of LCs enables the capability of dynamic Airy beams. Our proposed approach provides high precision, arbitrarily aligned LCs by specially designed metasurfaces, creating a unique platform for planar tunable photonic applications, for example 2D routers, 2D logic photonic circuits, etc.
